# How perceived goal attainment shapes student satisfaction: the chain mediating roles of competence acquisition, learning, and infrastructure

**DOI:** 10.3389/fpsyg.2025.1663142

**Published:** 2025-09-08

**Authors:** Chunshun Yan, Mengting Qian, Tianyi Sun

**Affiliations:** Department of Education Management, Faculty of Education, East China Normal University, Shanghai, China

**Keywords:** student satisfaction, perceived goal attainment, expectancy–disconfirmation theory, chain mediation model, higher education in China

## Abstract

**Introduction:**

Student satisfaction is a key indicator of higher education quality, yet the mechanisms linking students’ perceived goal attainment to their overall satisfaction remain underexplored. While expectancy–disconfirmation theory (EDT) has traditionally emphasized the gap between expected and actual outcomes, limited research has examined goal attainment as a personal cognitive benchmark within this framework. Drawing on EDT and self-determination theory, this study aimed to investigate how perceived goal attainment influences student satisfaction through the mediating roles of perceived competence acquisition and perceptions of learning and campus life.

**Methods:**

A cross-sectional survey was conducted among 2,981 students from 13 Chinese higher education institutions (HEIs). Participants completed validated scales measuring perceived goal attainment, competence acquisition, curriculum and learning, infrastructure and daily life, and satisfaction. Covariates included gender, grade, institution type, and regional development level. Structural equation modeling and bootstrapping (5,000 resamples) were used to test simple and chain mediation models.

**Results:**

Perceived goal attainment was positively correlated with satisfaction (*r* = 0.66, *p* < 0.001), competence acquisition (*r* = 0.66, *p* < 0.001), curriculum and learning (*r* = 0.48, *p* < 0.001), and infrastructure and daily life (*r* = 0.57, *p* < 0.001). In the model including curriculum and learning, the chain mediation path—Perceived Goal Attainment → Perceived Competence Acquisition → Curriculum and Learning → Student Satisfaction—was significant, with individual mediators also exerting partial effects. The total effect was 0.719, with 85.12% direct and 14.88% indirect effect (10.29% via competence, 1.67% via curriculum, 2.92% sequentially). In the model including infrastructure, the chained mediation pathway Perceived Goal Attainment → Perceived Competence Acquisition → Infrastructure and Daily Life → Student Satisfaction was also significant, with a 78.16% direct effect and 21.84% indirect effect (9.18% via competence, 8.62% via infrastructure, 4.03% sequentially).

**Conclusion:**

Perceived goal attainment influences student satisfaction both directly and indirectly via competence acquisition and improvements in academic and daily life experiences. By integrating EDT, SDT, and a chain mediation framework, the study highlights competence as a core psychological mechanism. HEIs should prioritize strategies that foster students’ sense of competence while optimizing curriculum and campus environments to enhance satisfaction and engagement.

## 1 Introduction

In the context of increasingly fierce global competition in higher education, student satisfaction has become a core indicator for measuring educational quality and institutional competitiveness (Wong and Chapman, 2023; Ikram and Kenayathulla, 2023). With the popularization of higher education, students have emerged as important stakeholders whose satisfaction evaluations directly impact institutional reputation and development (Gu and Lu, 2023). Therefore, understanding the formation mechanisms of student satisfaction is of great significance for improving educational quality and achieving sustainable development.

Expectancy-Disconfirmation Theory (EDT), as the dominant theoretical framework for explaining satisfaction formation, emphasizes that satisfaction stems from individuals’ comparison process between expectations and actual performance (Oliver, 1980). When actual performance meets or exceeds expectations, positive disconfirmation occurs, leading to satisfaction; conversely, it results in dissatisfaction (Spreng et al., 1996). This theory has been widely applied in higher education, providing a solid theoretical foundation for student satisfaction research (Zhang et al., 2022). Internationally, EDT-based studies have also been extended by integrating psychological needs theory, service quality models, and cross-cultural perspectives, showing that the interplay between expectation fulfillment, cognitive appraisal, and affective responses can vary substantially across educational systems (Gruber et al., 2010; Alves and Raposo, 2007; Wiers-Jenssen et al., 2002). As such, EDT offers the macro-level logic for this study.

In the Chinese higher education context, student satisfaction research holds particular significance. With a massive and increasingly diverse student body, higher education institutions (HEIs) face the challenge of meeting varied expectations and learning needs while maintaining high-quality educational experiences. Existing research indicates that Chinese students’ satisfaction is influenced by multiple factors including teaching quality, campus environment, and organizational management (Gu and Lu, 2023), yet there remains a gap in linking these contextual factors with internationally recognized determinants such as academic self-efficacy, perceived learning gains, and cognitive engagement, which have been shown in global studies to substantially mediate satisfaction outcomes (Douglas et al., 2015; Kandiko Howson and Mawer, 2013).

While EDT explains satisfaction through the gap between expectations and perceived performance, it does not, on its own, specify the psychological mechanisms that translate goal fulfillment into positive evaluations. To address this, the present study also draws on self-determination theory (SDT), which posits that satisfaction of basic psychological needs—particularly competence—enhances intrinsic motivation and well-being (Ryan and Deci, 2000). This perspective supports the inclusion of perceived competence acquisition as a mediating variable, linking goal attainment with broader satisfaction outcomes. SDT therefore provides the psychological driving mechanisms for this research.

Moreover, to capture the sequential and interrelated nature of mediators in the satisfaction formation process, this study incorporates a **c**hain mediation perspective. Chain mediation theory emphasizes that multiple mediators can operate in a logical sequence, where earlier mediators (e.g., competence acquisition) influence later ones (e.g., curriculum and learning or infrastructure and daily life), ultimately affecting the outcome variable (student satisfaction) (Taylor et al., 2008). This integration extends EDT by embedding it in a process-oriented model that reflects both psychological need fulfillment and the cumulative influence of interconnected experiential dimensions. This provides the structural mechanism through which EDT’s macro logic and SDT’s psychological drivers operate in practice.

This study focuses on the key question of how students’ Perceived Goal Attainment influences satisfaction. Perceived Goal Attainment reflects students’ subjective evaluation of the extent to which their educational expectations are realized, including knowledge and skill acquisition and personal capability enhancement (Elliott and Shin, 2002). Meanwhile, students’ Perceived Competence Acquisition, Curriculum and Learning, and Infrastructure and Daily Life may play important mediating roles in this process. Evidence from international research indicates that competence development is positively associated with both cognitive outcomes (e.g., critical thinking, problem-solving) and affective outcomes (e.g., academic satisfaction, well-being), and that the learning environment—including curriculum quality and campus infrastructure—can amplify these effects (Kuh et al., 2006; Trowler, 2010). Grounded in SDT, competence is conceptualized as a basic psychological need; when this need is fulfilled, students are more likely to evaluate their curriculum, learning environment, and infrastructure positively and report higher satisfaction (Ryan and Deci, 2000).

Based on chain mediation theory, this study proposes two sequential mediation pathways: Perceived Goal Attainment → Perceived Competence Acquisition → Curriculum and Learning → Student Satisfaction and Perceived Goal Attainment → Perceived Competence Acquisition → Infrastructure and Daily Life → Student Satisfaction. This mechanisms not only enrich the application of expectancy-disconfirmation theory but also provide a new perspective for understanding the complex formation process of student satisfaction.

Therefore, based on the expectancy-disconfirmation theoretical framework, this study explores the influence mechanism of perceived goal attainment on student satisfaction and the mediating roles of perceived competence acquisition, curriculum and learning, and infrastructure and daily life through a survey of 2,981 undergraduate students from 13 HEIs in China. By incorporating both domestic and international perspectives, the study aims to bridge theoretical and contextual gaps, offering a more comprehensive understanding of satisfaction formation mechanisms applicable to diverse higher education systems. The research findings will provide scientific evidence for higher education institutions to develop strategies for enhancing student satisfaction.

## 2 Literature review

### 2.1 Expectancy–disconfirmation theory (EDT) and its application to student satisfaction

Student satisfaction has long been conceptualized as analogous to customer satisfaction in consumer behavior research (Elliott and Healy, 2001; Brown and Mazzarol, 2009). Among various frameworks, the expectancy–performance disconfirmation theory (EDT) developed by Olshavsky and Miller (1972) and extended by Oliver (1980, 2010) remains one of the most influential explanations for how satisfaction forms. Oliver (2010) described satisfaction as a “fulfillment response,” a judgment that perceived performance provides a pleasurable level of fulfillment. This classic view emphasizes two core ideas: satisfaction is an evaluative attitude directed at a specific target, and it depends on the extent to which perceived performance meets or exceeds initial expectations (Oliver, 1980; Oliver and Burke, 1999).

EDT posits that satisfaction arises from comparing expectations with perceived performance: positive disconfirmation occurs when performance exceeds expectations, leading to satisfaction, while negative disconfirmation results when performance falls short (Spreng et al., 1996). The theory distinguishes key constructs such as expectations, perceived performance, perceived value, disconfirmation, and outcomes like loyalty and complaints (Zeithaml, 1988; Heidenreich et al., 2015). Research has explored both direct effects of expectations and performance (Anderson, 1973; Cardozo, 1965; Evangelidis and Van Osselaer, 2018) and the mediating role of disconfirmation as a distinct construct (Niedrich et al., 2005; Spreng and Page, 2003).

Beyond consumer contexts, EDT has been adapted to public services and citizen satisfaction (Van Ryzin, 2004, 2006, 2013). Its focus on internal cognitive comparisons—how expectations and perceived performance jointly shape satisfaction—has also been foundational for evaluating service quality and institutional effectiveness in higher education (Elliott and Shin, 2002).

Recent empirical work reaffirms EDT’s relevance across diverse university contexts. For example, Khan and Hemsley-Brown (2024) found that UK students’ satisfaction with tuition costs and institutional reputation depended strongly on expectation disconfirmation. Alzahrani and Seth (2021) showed that meeting expected information and service quality shaped British students’ satisfaction with emergency online learning during COVID-19. Similarly, Surya Bahadur et al. (2024) demonstrated among Indian and Nepalese business students that program quality and administrative services that exceeded expectations boosted satisfaction, while gaps in teacher quality did not. In Bangladesh, Umar and Hasan (2024) found that strong support services aligned with expectations enhanced satisfaction in engineering students.

Guided by this theoretical lens, the present study not only applies EDT but also integrates complementary frameworks to better capture the proposed mediation process. Specifically, self-determination theory (SDT) (Ryan and Deci, 2000) highlights competence as a core psychological need whose satisfaction directly contributes to positive affective evaluations such as satisfaction. This aligns with our conceptualization of “Perceived Competence Acquisition” as a mediator. Furthermore, the chain mediation perspective (Taylor et al., 2008; Hayes, 2013) emphasizes that causal mechanisms can unfold through multiple, sequential mediators, offering a theoretical basis for our hypothesis that competence acquisition influences satisfaction indirectly via enhanced perceptions of learning and campus life.

In this integrated framework, satisfaction is conceptualized as shaped by how well expectations are fulfilled across four dimensions: perceived goal attainment, perceived competence acquisition, curriculum and learning, and infrastructure and daily life. The model tests whether perceived goal attainment influences satisfaction both directly and indirectly through competence acquisition and these two experience-based mediators, responding to calls for a more nuanced understanding of how institutional factors and individual evaluations interact in Chinese higher education.

### 2.2 Student satisfaction in the Chinese higher education context

Student satisfaction is generally defined as students’ subjective evaluation of their learning experiences and the perceived value of the education they receive (Elliott and Shin, 2002; Douglas et al., 2006). In line with EDT, student satisfaction is shaped by the interaction between students’ initial expectations and their actual experiences within the university environment (Appleton-Knapp and Krentler, 2006; Alves and Raposo, 2007).

International research has established that student satisfaction is a multi-dimensional construct that encompasses various aspects of academic and campus life, including teaching quality, curriculum relevance, faculty–student interaction, support services, and campus facilities (Gruber et al., 2010; Soomro et al., 2020; Kanwar and Sanjeeva, 2022; Ikram and Kenayathulla, 2023; Dugenio-Nadela et al., 2023). For example, Douglas et al. (2006) developed a comprehensive student satisfaction model that includes dimensions such as course content, teaching methods, assessment, learning resources, and campus environment. Similarly, Letcher and Neves (2010) found that students’ perceptions of the academic experience, including faculty effectiveness and learning resources, significantly predict overall satisfaction.

In the Chinese context, studies have adapted these frameworks to local cultural and institutional settings. Zhang and Yue (2009) developed an evaluation system covering teaching content, student engagement, extracurricular activities, and living services. Li (2017) advanced this work by constructing a higher education satisfaction index model that integrates talent cultivation, perceived management, campus culture, perceived quality, and infrastructure support.

A recent synthesis by Gu and Lu (2023) reviewed 48 studies and identified seven major dimensions influencing Chinese students’ satisfaction: teaching quality, school reputation, campus environment, organizational management, logistical support (e.g., housing and dining), personal improvement (self-development), and financial aspects. Teaching quality, campus environment, administrative management, and logistical services were identified as the most critical factors. Despite this progress, prior research still focus on descriptive measurement rather than exploring how these factors interact or mediate one another (Zhang and Yue, 2009).

There is a clear need for more nuanced analyses that reveal the process mechanisms behind student satisfaction, aligned with international models that highlight multi-step or chain mediation pathways (Cardona and Bravo, 2012). By addressing this gap, the present study seeks to deepen understanding of how students’ expectations, perceived competence acquisition, and perceptions of curriculum and learning as well as infrastructure and daily life work together to shape satisfaction in the Chinese higher education system.

### 2.3 Perceived goal attainment and student satisfaction

The relationship between students’ initial expectations and their satisfaction has long been a central focus in higher education research, largely inspired by classical customer satisfaction theory (Olshavsky and Miller, 1972; Oliver, 1980). When institutional performance meets or exceeds expectations, satisfaction tends to increase; when it falls short, dissatisfaction results (Spreng et al., 1996). While institutional quality may be objectively stable, students’ subjective expectations often play a decisive role in shaping satisfaction levels (Appleton-Knapp and Krentler, 2006). Studies have shown that unrealistically high expectations can lower satisfaction (Brown et al., 2008), whereas more realistic and attainable goals are linked to higher satisfaction in both Western and Chinese contexts (Zhang and Lin, 2014).

In response to these findings, recent studies have highlighted the need to measure not only expectations but also the extent to which students perceive their goals as being fulfilled through the educational process (Douglas et al., 2006; Alves and Raposo, 2007; Soomro et al., 2020; Gabrah et al., 2023; Maniriho, 2024). This construct, known as Perceived Goal Attainment, reflects students’ subjective judgment of how effectively the university’s educational activities contribute to their knowledge, skills, and personal development in line with their initial expectations (Elliott and Shin, 2002; Gruber et al., 2010). Research in various higher education settings has shown that when students believe their academic and personal goals are being met, they are more likely to report higher levels of overall satisfaction (Elliott and Shin, 2002; Douglas et al., 2006).

By bridging students’ expectations and the perceived effectiveness of educational experiences, perceived goal attainment plays a critical mediating role in explaining how satisfaction is formed (Letcher and Neves, 2010; Gruber et al., 2010). For instance, aligning students’ expectations with actual educational value—such as tuition fees or academic outcomes—can reduce the “expectation gap” and increase satisfaction (Khan and Hemsley-Brown, 2021). Beyond EDT, SDT (Ryan and Deci, 2000) suggests that attaining valued goals satisfies the basic psychological need for competence, thereby fostering intrinsic motivation, positive affect, and stronger commitment to the learning process. This dual-theoretical perspective indicates that goal attainment can enhance satisfaction both as a cognitive evaluation (meeting expectations) and as a psychological fulfillment (meeting needs), and that this process may extend to students’ perceptions of curriculum and learning as well as infrastructure and daily life.

### 2.4 The mediating role of perceived competence acquisition

In higher education research, Perceived Competence Acquisition refers to students’ subjective evaluation of the extent to which they believe they have developed relevant knowledge, skills, and capacities during their university experience (Komarraju et al., 2010; Cazan and Schiopca, 2014; Bustos-Usta et al., 2024). This concept builds on the view that fulfilling students’ higher-order needs for self-development and mastery is a key driver of satisfaction and positive outcomes (Maslow, 1954; Ryan and Deci, 2000). According to SDT, the need for competence is an essential psychological driver that motivates students to engage with learning environments and shapes their subsequent satisfaction and well-being (Ryan and Deci, 2000; Fryer and Elliot, 2008).

From a goal-setting perspective, goal attainment provides feedback signaling successful task performance and mastery development (Locke and Latham, 2002). When students perceive that they have met or exceeded their academic and personal development goals, they infer that they have acquired or strengthened relevant competencies (Bandura, 1997). Social cognitive theory similarly posits that achieving self-set goals enhances self-efficacy and perceived competence through positive reinforcement and mastery experiences (Bandura, 1997; Schunk and Pajares, 2009). In other words, goal fulfillment acts as evidence that students possess the skills and abilities needed to succeed, which reinforces their competence beliefs (Elliot and Dweck, 2005).

From a psychoanalytic perspective, Adler emphasized that an individual’s social needs and perceived competence development are crucial for personality growth and overall well-being (Adler, 1927). In the Chinese context, Wang and Liu (2019) similarly highlighted that perceived gains in competence reflect the degree to which students’ social and psychological needs are met through the educational process. As students perceive their competence to grow, they are more likely to experience positive emotions, which in turn shape their evaluation of the learning environment (Bastian et al., 2014). This process also resonates with Conservation of Resources theory, which suggests that goal attainment signals a gain in personal resources (e.g., skills, knowledge), reducing stress and enabling further resource accumulation such as confidence and competence (Hobfoll, 1989).

Empirical studies support the importance of perceived competence as an intermediate mechanism connecting institutional performance to students’ subjective satisfaction. For example, Komarraju et al. (2010) found that university environments that encourage skill development and academic competence enhance students’ intrinsic motivation and satisfaction. Similarly, Cazan and Schiopca (2014) showed that students who feel they have acquired meaningful competencies during their studies report greater academic engagement and satisfaction. International evidence has also demonstrated that perceived competence is positively linked with students’ evaluation of their overall university experience (Bastian et al., 2014; Komarraju et al., 2010). Yang et al. (2022) further found that students’ perceptions of goal fulfillment in learning contexts directly predicted competence beliefs, which subsequently mediated their academic satisfaction. In fact, satisfying the need for competence has been empirically linked to greater student well-being and contentment in school. Ryan and Deci (2020) noted that students who experience higher competence fulfillment tend to report higher overall satisfaction with their educational experience. This aligns with a broad body of work indicating that when academic environments provide optimal challenges, feedback, and opportunities to build mastery, students’ sense of competence increases, leading to more positive appraisals of their learning outcomes.

Drawing on the EDT and Maslow’s hierarchy of needs, this study conceptualizes perceived competence acquisition as a key mediator between perceived goal attainment and other dimensions of student experience. Specifically, when students believe their expectations for learning and skill development are fulfilled, they are more likely to perceive gains in competence, which in turn positively influence how they evaluate their broader learning and campus life (Komarraju et al., 2010; Cazan and Schiopca, 2014). Recent studies have also found that perceived competence functions as a significant mediator linking students’ goal achievement and their academic or life satisfaction (Van Dinther et al., 2011; Yang et al., 2022). This perspective aligns with prior research showing that students’ positive emotional states and self-perceived mastery enhance their satisfaction with learning contexts.

### 2.5 The mediating role of perceived curriculum and learning

Perceived curriculum and learning refer to students’ evaluative judgments of their academic experiences, including course content, instructional quality, classroom interaction, assessment formats, and feedback mechanisms (Gruber et al., 2010; Suarman et al., 2013). These factors represent the core of the academic process and directly shape students’ cognitive and emotional responses toward their educational environment. Studies have consistently shown that high-quality teaching and intellectually engaging curricula are major predictors of student satisfaction (Aldemir and Gulcan, 2004; Alves and Raposo, 2007; Bell, 2021).

Drawing on EDT, students develop expectations about what and how they will learn in HEIs. When actual academic experiences align with or exceed these expectations—such as receiving timely feedback, experiencing curriculum relevance, or perceiving fairness in assessment—positive disconfirmation leads to enhanced satisfaction (Oliver, 1980; Elliott and Shin, 2002).

In the Chinese context, where academic success remains a dominant performance indicator, satisfaction with learning is particularly salient. For instance, Zhang et al. (2013) and Gu and Lu (2023) emphasize that perceptions of teaching quality, assessment fairness, and classroom engagement are central to Chinese students’ satisfaction formation. Accordingly, perceived curriculum and learning are hypothesized to function not only as a direct contributor to student satisfaction but also as a mediating mechanism between students’ perceived goal attainment and their overall evaluation of HEIs life.

### 2.6 The mediating role of perceived infrastructure and daily life

Perceived infrastructure and daily life refer to students’ evaluations of the material and logistical aspects of their HEIs environment, including dormitory quality, food service, transportation, campus safety, internet access, and other support facilities (Douglas et al., 2006; Gruber et al., 2010). These dimensions are especially important in shaping students’ sense of well-being and institutional attachment, particularly for residential and full-time students.

Under the framework of EDT, students enter HEIs with expectations not only about academic experiences but also about the surrounding living conditions. If the actual living environment—such as dormitory comfort, service affordability, campus safety, or reliability of internet infrastructure—meets or surpasses these expectations, positive disconfirmation occurs, leading to stronger emotional attachment to the institution and higher satisfaction (Oliver, 1980; Tessema et al., 2012). International research supports this view: Arambewela and Hall (2009) found that infrastructure and safety significantly predict international students’ satisfaction. Similarly, Suarman et al. (2013) and Jiménez-Bucarey et al. (2021) showed that university service quality and facility management play a decisive role in overall satisfaction.

In the Chinese higher education context, students’ satisfaction is also highly responsive to infrastructure-related factors such as dormitory conditions, affordability of campus services, and reliability of technical infrastructure (Gu and Lu, 2023).

### 2.7 The chain mediating role of perceived competence acquisition and two campus dimensions

Recent research suggests that competence acquisition (i.e., the belief in one’s learning progress and skill development) may serve as a precursor to improved perceptions of both academic and non-academic campus experiences (Komarraju et al., 2010; Nguyen et al., 2024). In particular, as students develop a stronger sense of competence, they tend to engage more actively in learning processes and participate more confidently in campus life, which in turn enhances their perception of teaching quality, service delivery, and infrastructure effectiveness (Van Dinther et al., 2011).

From the perspective of SDT, competence fulfillment not only generates intrinsic motivation but also positively influences the way students interpret subsequent experiences. Luna-Cortes (2024) further illustrates this chain process, finding that students’ cognitive recalibration (e.g., adjusting expectations and recognizing competence growth) improved their satisfaction through indirect changes in experience appraisal. Analogously, we propose that competence acquisition may sequentially influence satisfaction by enhancing students’ perceptions of both curriculum and learning, and infrastructure and daily life.

**Figure 1:**
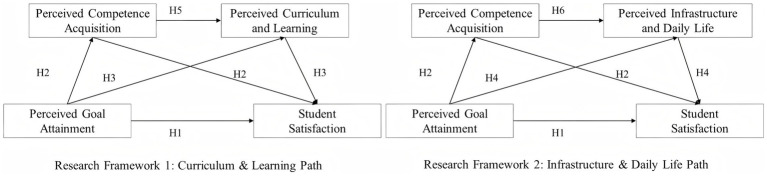
Research framework.

By integrating these perspectives, the proposed model links cognitive evaluation (EDT), psychological needs (SDT), and structural pathway mechanisms (chain mediation) into a coherent explanatory chain from perceived goal attainment to overall satisfaction. Accordingly, this study develops a chain-mediated hypothetical model (Figure 1) to clarify the relationships among perceived goal attainment, perceived competence acquisition, curriculum and learning, infrastructure and daily life, and student satisfaction, and proposes the following hypotheses:

*H1:* Perceived goal attainment is significantly and positively related to student satisfaction.
*H2:* Perceived competence acquisition significantly mediates the relationship between perceived goal attainment and student satisfaction.
*H3:* Perceived curriculum and learning significantly mediates the relationship between perceived goal attainment and student satisfaction.
*H4:* Perceived infrastructure and daily life significantly mediates the relationship between perceived goal attainment and student satisfaction.
*H5:* Perceived competence acquisition and perceived curriculum and learning significantly and sequentially mediate the relationship between perceived goal attainment and student satisfaction.
*H6:* Perceived competence acquisition and perceived infrastructure and daily life significantly and sequentially mediate the relationship between perceived goal attainment and student satisfaction.

## 3 Methodology

### 3.1 Participants

Between April and June 2021, to ensure the representativeness of the data, a stratified sampling strategy was adopted, taking into account both institutional level and institutional quality. Participants were recruited from 13 HEIs in Zhejiang, Jiangsu, Guizhou, and other provinces across China. Data were collected through online surveys using Questionnaire Star, WeChat, and other digital platforms. A total of 3,819 responses were initially obtained. All participants were recruited on a voluntary basis through convenience sampling. The survey introduction provided an overview of the research purpose, eligibility criteria, confidentiality assurances, and participants’ right to withdraw at any time. Prior to data collection, the research procedures and instruments were reviewed and approved by the institutional ethics committee in accordance with standard ethical guidelines. After removing invalid and low-quality responses (e.g., patterned responses or completion times under 180 s), a final valid sample of 2,981 responses was retained, yielding a valid response rate of 78.06%. The final sample comprised 38.1% male and 61.9% female respondents. The majority were Han Chinese (89.4%), with ethnic minorities accounting for 10.6%. The sample included first- to fifth-year undergraduates (56.8, 19.8, 17.4, 5.6, and 0.4%, respectively), which reflects the typical four-year undergraduate structure in China with some five-year programs. Regarding disciplinary distribution, 34.49% majored in science and engineering, 13.79% in medical science, 46.29% in economics, management, and humanities, and 4.43% in arts and physical education. Regarding regional distribution, 53.7% of students were from HEIs in more economically developed regions, and 46.3% from less developed regions. By institutional type, 82.2% were ordinary undergraduate students enrolled in ordinary undergraduate universities, while 17.8% were vocational undergraduate students enrolled in vocational undergraduate institutions. This distribution aligns with the national pattern in which the number of ordinary undergraduate students substantially exceeds that of vocational undergraduates.

### 3.2 Measures

#### 3.2.1 Students’ perceived goal attainment

The measurement of students’ perceived goal attainment was initially developed through expert focus group discussions and a comprehensive review of relevant literature. The preliminary version of the scale consisted of five items covering students’ expectations for various aspects of HEIs life. An initial confirmatory factor analysis (CFA) indicated that the measurement model required improvement, with fit indices showing GFI = 0.959, AGFI = 0.878, and RMSEA = 0.139. In line with established recommendations, items with low factor loadings or conceptual overlap were removed. Specifically, two items—“My university meets my expectations for campus life” and “I can achieve my established goals at my university”—were deleted based on factor loadings and expert consensus.

The final version of the scale retained 3 items: “My university meets my expectations for academic learning,” “My university meets my expectations for skill development,” and “My university meets my expectations for social life.” Each item was rated on a five-point Likert scale ranging from “1 = strongly disagree” to “5 = strongly agree,” with higher scores indicating greater levels of perceived goal attainment. Due to the small number of indicators, the CFA model degrees of freedom were limited; therefore, the construct validity was primarily evaluated using standardized factor loadings, composite reliability (CR), and average variance extracted (AVE). The standardized factor loadings were 0.92, 0.96, and 0.92, respectively, with corresponding squared multiple correlations (*R^2^*) of 0.851, 0.919, and 0.838. The computed CR was 0.95 (>0.70) (Hair et al., 2018) and the AVE was 0.87 (>0.50) (Fornell and Larcker, 1981), demonstrating satisfactory convergent validity. Moreover, the scale exhibited excellent internal consistency, with a Cronbach’s alpha of 0.95.

#### 3.2.2 Perceived competence acquisition

Perceived competence acquisition was measured based on the scale developed by Wang and Guo (2020) and refined through expert focus group discussions. The scale consists of four items, including statements such as “My independent learning ability has been enhanced,” “My knowledge base and perspectives have been broadened,” “My ability to identify and solve problems has been improved,” and “My employability has been strengthened.” Participants rated the items on a five-point Likert scale, ranging from “1 = strongly disagree” to “5 = strongly agree,” with higher scores indicating higher levels of perceived competence acquisition. The results of the confirmatory factor analysis (CFA) indicated an acceptable model fit. Given the large sample size (N = 2,981), the chi-square statistic (χ^2^ = 24.93, df = 2, χ^2^/df = 12.46) were not emphasized in evaluating model fit, as it is prone to inflation in large-sample contexts with low degrees of freedom (Byrne, 2010), whereas alternative fit indices indicated excellent model fit (GFI = 0.996, AGFI = 0.979, RMSEA = 0.062). The scale also demonstrated strong internal consistency reliability, with a Cronbach’s alpha coefficient of 0.94.

#### 3.2.3 Perceived curriculum and learning

Perceived curriculum and learning was assessed using a scale adapted from Hu (2018) and Bao (2014) and further refined through expert focus group discussions. Participants rated the items on a five-point Likert scale, ranging from “1 = strongly disagree” to “5 = strongly agree,” with higher scores indicating higher levels of perceived satisfaction with curriculum and learning. The “Curriculum and Learning” subscale includes five items, such as “The assessment format of courses is reasonable,” “Course content is rich and practical,” and “Teachers provide timely feedback on assignments.” The confirmatory factor analysis results for this dimension indicated acceptable model fit, with GFI = 0.990, AGFI = 0.969, and RMSEA = 0.072. The Cronbach’s alpha for this dimension was 0.94.

#### 3.2.4 Perceived infrastructure and daily life

Perceived infrastructure and daily life was assessed using a scale adapted from Xu (2017) and Bao (2014), refined via expert input. Participants responded to each item using a five-point Likert scale ranging from 1 (strongly disagree) to 5 (strongly agree), with higher scores indicating greater satisfaction with infrastructure and daily life. The final version retained three items: “Comfortable dormitory environment with adequate living facilities,” “Reliable campus utilities (water, electricity, internet) supporting academic and living needs,” and “Comprehensive recreational and living facilities enhancing student experience.” All items used the same five-point Likert scale. Standardized factor loadings were 0.76, 0.83, and 0.80, with *R^2^* values of 0.575, 0.692, and 0.628. The CR was 0.84 (>0.70) and the AVE was 0.63 (>0.50), indicating satisfactory convergent validity. Additionally, the Cronbach’s alpha for the scale was 0.95, indicating excellent reliability.

#### 3.2.5 Student satisfaction

Student satisfaction was assessed using the scale developed by Chadwick and Ward (1987), which evaluates graduate attitudes about the cost and value of the education and services received while in college. The scale consists of three items, including statements such as “If I had money, I would donate to my university,” “If I were the head of a company, I would prioritize hiring graduates from my university,” and “I would recommend my university to others.” Participants rated their level of satisfaction using a five-point Likert scale, ranging from “1 = strongly disagree” to “5 = strongly agree,” with higher scores indicating greater satisfaction with their university experience.

The results of the confirmatory factor analysis (CFA) indicated good scale validity, with standardized factor loadings of 0.83, 0.91, and 0.90 for the three items. The squared multiple correlations (*R^2^*) were 0.690, 0.821 and 0.805. The composite reliability (CR) for the scale was calculated to be 0.91, exceeding the recommended threshold of 0.70, indicating good internal consistency. The average variance extracted (AVE) was 0.77, which also exceeds the threshold of 0.50, demonstrating strong convergent validity. Additionally, the Cronbach’s alpha for the scale was 0.91, indicating excellent reliability.

#### 3.2.6 Control variables

Previous studies suggest that demographic characteristics can influence student satisfaction. In this study, gender, academic year, institution type, regional development level were included as control variables in the model.

### 3.3 Data analysis

The data were analyzed using SPSS 27.0 and AMOS 28.0. First, confirmatory factor analysis and reliability analysis were conducted on each scale using SPSS 27.0 and AMOS 28.0 to assess their validity and reliability. Second, a common method bias test, descriptive statistics, and correlation analyses were performed using SPSS 27.0. Third, Model 6 of the PROCESS macro was used to examine the direct and indirect relationships among Perceived Goal Attainment, Student Satisfaction, Perceived Competence Acquisition, Perceived Curriculum and Learning, and Perceived Infrastructure and Daily Life. Additionally, the mediating effect of work alienation was further assessed using the bias-corrected bootstrap method. If the 95% confidence interval (CI) for the mediating effect does not include zero, it indicates the establishment of a significant mediating role.

## 4 Results

### 4.1 Exploratory factor analysis

To ensure the reliability and validity of the measurement instruments, the dataset was randomly split into two halves. An exploratory factor analysis (EFA) was conducted on the first half of the data (*n* = 1,491) using principal components analysis with Varimax rotation. The results demonstrated that the Cronbach’s *α* coefficients for all dimensions ranged from 0.75 to 0.92, indicating satisfactory internal consistency. The composite reliability (CR) values ranged from 0.86 to 0.92, exceeding the recommended threshold of 0.70 (Hair, 1998). The average variance extracted (AVE) values ranged from 0.67 to 0.79, meeting or exceeding the recommended minimum standard of 0.50 (Fornell and Larcker, 1981). These results indicate that the scales exhibit acceptable reliability and convergent validity.

### 4.2 Confirmatory factor analysis

Subsequently, a confirmatory factor analysis (CFA) was conducted on the remaining half of the data (*n* = 1,490). The Curriculum and Learning scale and the Infrastructure and Daily Life scale were tested separately in the CFA model because the standardized factor loading of one item in each dimension was relatively low (below 0.50). For the Curriculum and Learning, the model fit indices were χ^2^/df = 5.048, RMSEA = 0.052, GFI = 0.962, AGFI = 0.947, NFI = 0.972, IFI = 0.978, TLI = 0.972, CFI = 0.978, and PNFI = 0.787. For the Infrastructure and Daily Life, the CFA results indicated χ^2^/df = 6.140, RMSEA = 0.059, GFI = 0.962, AGFI = 0.942, NFI = 0.969, IFI = 0.974, TLI = 0.966, CFI = 0.974, and PNFI = 0.745. Chi-square values tend to be inflated in large samples (Byrne, 2010). Overall, these results indicate that the questionnaire demonstrates good structural validity.

### 4.3 Common method bias test and multicollinearity diagnostics

To assess potential common method bias (CMB), three complementary techniques were applied. First, Harman’s single-factor test indicated that 11 factors with eigenvalues above 1.0 were extracted, and the first factor accounted for 33.10% of the total variance. When the proportion of variance explained by the first factor is below 40%, it is generally interpreted as evidence that no substantial common method bias exists (Fuller et al., 2016; Podsakoff et al., 2003).

Second, a single-factor confirmatory factor analysis was performed. The single-factor model showed poor fit (*χ*^2^/df = 149.61, CFI = 0.591, RMSEA = 0.223), indicating that a single factor cannot adequately represent all measurement items. Following Podsakoff et al. (2003), poor fit in a single-factor CFA suggests that common method bias is unlikely to be a serious concern.

Third, a Common Latent Factor (CLF) approach was applied as a robustness check. A latent method factor was introduced, loading on all observed indicators, and model fit indices were compared between models with and without the CLF. For the learning model, adding the CLF improved the fit slightly (CFI: 0.984 → 0.994, ΔCFI = 0.010; RMSEA: 0.054 → 0.035, ΔRMSEA = 0.019). For the living model, the changes were similarly small (CFI: 0.980 → 0.988, ΔCFI = 0.008; RMSEA: 0.063 → 0.056, ΔRMSEA = 0.007). For large group sizes, Rutkowski and Svetina (2014) recommended more liberal criteria for assessing model fit changes, namely ΔCFI ≤ 0.020 and ΔRMSEA ≤ 0.030. The present results met these thresholds for both models, suggesting that substantial common method variance is unlikely to exist.

Additionally, to ensure the absence of multicollinearity, variance inflation factors (VIFs) were calculated for all independent variables. The VIF values ranged from 1.57 to 2.16, and tolerance values ranged from 0.46 to 0.64, which are well within the commonly accepted standards of VIF < 5 and tolerance > 0.20, indicating no evidence of problematic multicollinearity (Hair et al., 2018; Menard, 1995).

### 4.4 Descriptive statistics and correlation analysis

Preliminary checks indicated that skewness values for all continuous variables ranged from −0.53 to 0.44, and kurtosis values ranged from 0.08 to 1.16, falling within the acceptable range of −2 to +2 (Kline, 2015), suggesting approximate normality of the data distribution. The results of the correlation analysis (see Table 1) indicate that Perceived Goal Attainment was significantly and positively correlated with Student Satisfaction (*r* = 0.66, *p* < 0.001), Perceived Competence Acquisition (*r* = 0.66, *p* < 0.001), Curriculum and Learning (*r* = 0.48, *p* < 0.001), and Infrastructure and Daily Life (*r* = 0.53, *p* < 0.001). Student Satisfaction was also significantly and positively related to Perceived Competence Acquisition (*r* = 0.50, *p* < 0.001), Curriculum and Learning (*r* = 0.39, *p* < 0.001), and Infrastructure and Daily Life (*r* = 0.47, *p* < 0.001). Additionally, Perceived Competence Acquisition was significantly and positively associated with both Curriculum and Learning (*r* = 0.58, *p* < 0.001) and Infrastructure and Daily Life (*r* = 0.50, *p* < 0.001). Finally, Curriculum and Learning was significantly and positively correlated with Infrastructure and Daily Life (*r* = 0.42, *p* < 0.001). These findings provide initial support for the proposed relationships among the study variables.

**Table 1:** Descriptive statistics and correlation analysis (*N* = 2,981).

Variable	M	SD	1	2	3	4	5
1 = Perceived goal attainment	3.27	0.70	1				
2 = Student satisfaction	3.19	0.77	0.66^***^	1			
3 = Perceived competence acquisition	3.46	0.61	0.66^***^	0.50^***^	1		
4 = Curriculum and learning	3.65	0.67	0.48^***^	0.39^***^	0.58^***^	1	
5 = Infrastructure and daily life	3.24	0.75	0.53^***^	0.47^***^	0.50^***^	0.42^***^	1

*p < 0.05, **p < 0.01, ***p < 0.001 (two-tailed); M, mean; SD, standard deviation.

### 4.5 Mediation effect analysis

#### 4.5.1 Testing the mediating effect of curriculum and learning

The regression analysis results are presented in Table 2. After controlling for gender, grade, institution and regional variables, Perceived Goal Attainment showed a significant positive direct effect on Student Satisfaction (*β* = 0.72, *p* < 0.001), providing support for H1. In addition, Perceived Goal Attainment had a significant positive effect on Perceived Competence Acquisition (*β* = 0.57, *p* < 0.001) and Curriculum and Learning (*β* = 0.16, *p* < 0.001), indicating that the prerequisite conditions for testing the mediation hypotheses involving Perceived Competence Acquisition and Curriculum and Learning were met.

**Table 2:** Regression analysis for the mediation effects of curriculum and learning (*N* = 2,981).

Outcome variables	Independent variables	*R*	*R* ^2^	*F*	*β*	SE	t	*p*
Student satisfaction		0.67	0.45	489.59***				
	Gender				0.01	0.02	0.54	0.591
	Grade				−0.03	0.01	−2.97**	0.003
	Institution				0.06	0.03	2.15*	0.032
	Regional				0.18	0.02	8.16***	0.000
	Perceived goal attainment				0.72	0.02	47.57***	0.000
Perceived competence acquisition		0.67	0.45	483.25***				
	Gender				−0.03	0.02	−1.78	0.075
	Grade				0.05	0.01	5.77***	0.000
	Institution				0.01	0.02	0.49	0.621
	Regional				−0.06	0.02	−3.35***	0.000
	Perceived goal attainment				0.57	0.01	47.37***	0.000
Curriculum and learning		0.60	0.37	283.86***				
	Gender				0.04	0.02	1.93	0.054
	Grade				0.09	0.01	8.23***	0.000
	Institution				0.02	0.03	0.88	0.377
	Regional				0.07	0.02	3.21**	0.001
	Perceived goal attainment				0.16	0.02	8.57***	0.000
	Perceived competence acquisition				0.48	0.02	22.49***	0.000
Student satisfaction		0.68	0.46	367.40***				
	Gender				0.01	0.02	0.64	0.522
	Grade				−0.05	0.01	−4.30***	0.000
	Institution				0.06	0.03	2.04*	0.041
	Regional				0.19	0.02	8.43***	0.000
	Perceived goal attainment				0.61	0.02	30.51***	0.000
	Perceived competence acquisition				0.13	0.02	5.23***	0.000
	Curriculum and learning				0.08	0.02	3.95***	0.000

*p < 0.05, **p < 0.01, ***p < 0.001 (two-tailed), Gender was coded as 1 = male and 2 = female; Grade ranged from 1 = first year to 5 = fifth year; Institution was coded as 1 = undergraduate universities and 2 = vocational undergraduate institutions; Regional was coded as 1 = developed areas and 2 = underdeveloped areas, similarly hereinafter.

**Figure 2:**
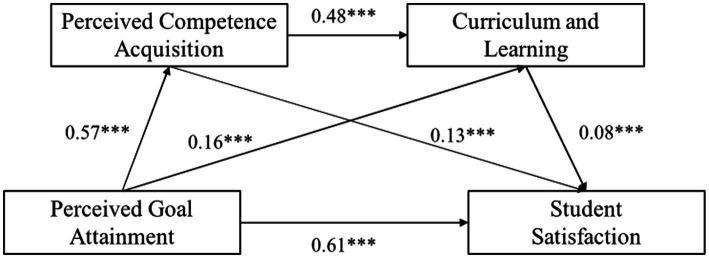
Chain mediation model linking perceived goal attainment, perceived competence acquisition, curriculum and learning, and student satisfaction. Standardized path coefficients are reported. All paths are significant at *p* < 0.001 unless otherwise indicated, similarly hereinafter.

As shown in Table 3 and Figure 2, both Perceived Competence Acquisition and Curriculum and Learning were positively associated with Student Satisfaction (*β* = 0.13, *p* < 0 0.001; *β* = 0.08, *p* < 0.001), lending support to H2 and H3. Perceived Competence Acquisition was also positively associated with Curriculum and Learning (*β* = 0.48, *p* < 0.001), consistent with the sequential mediation proposed in H5.

**Table 3:** Direct and indirect effects of perceived goal attainment on student satisfaction via perceived competence acquisition and curriculum and learning.

Path	Effect	SE	Bias-corrected 95%CI		Ratio
			Lower	Upper	
Total effect	0.719	0.015	0.689	0.749	100%
Direct effect	0.612	0.020	0.572	0.651	85.12%
Total indirect effect	0.107	0.019	0.071	0.146	14.88%
Indirect path1	0.074	0.020	0.035	0.114	10.29%
Indirect path2	0.012	0.004	0.005	0.022	1.67%
Indirect path3	0.021	0.006	0.010	0.034	2.92%

Indirect path 1: Perceived Goal Attainment → Perceived Competence Acquisition → Student Satisfaction; Indirect path 2: Perceived Goal Attainment → Curriculum and Learning → Student Satisfaction; Indirect path 3: Perceived Goal Attainment → Perceived Competence Acquisition → Curriculum and Learning → Student Satisfaction.

The total effect of Perceived Goal Attainment on Student Satisfaction was 0.719, with the direct effect accounting for 85.12% (95% CI = [0.572, 0.651]) and the total indirect effect accounting for 14.88% (effect = 0.107, 95% CI = [0.071, 0.146]). Specifically, the indirect effect of the first path “Perceived Goal Attainment → Perceived Competence Acquisition → Student Satisfaction” (Indirect Path 1) was 0.074 (95% CI = [0.035, 0.114]), accounting for 10.29% of the total effect. The indirect effect of the second path “Perceived Goal Attainment → Curriculum and Learning → Student Satisfaction” (Indirect Path 2) was 0.012 (95% CI = [0.005, 0.022]), accounting for 1.67% of the total effect. The indirect effect of the third path “Perceived Goal Attainment → Perceived Competence Acquisition → Curriculum and Learning → Student Satisfaction” (Indirect Path 3) was 0.021 (95% CI = [0.010, 0.034]), accounting for 2.92% of the total effect. The total indirect effect was 0.107 (95% CI = [0.071, 0.146]), indicating that the overall mediation effect path was significant.

#### 4.5.2 Testing the mediating effect of infrastructure and daily life

The regression analysis results are presented in Table 4. After controlling for gender, grade level, institution type, and regional development level, Perceived Goal Attainment had a significant positive direct effect on Student Satisfaction (*β* = 0.72, *p* < 0.001); see Table 2 supporting H1. In addition, Perceived Goal Attainment significantly predicted both Perceived Competence Acquisition (*β* = 0.57, *p* < 0.001; see Table 2) and Infrastructure and Daily Life (*β* = 0.40, *p* < 0.001).

**Table 4:** Regression analysis for the mediation effects of infrastructure and daily life (*N* = 2,981).

Outcome variables	Independent variables	*R*	*R* ^2^	*F*	*β*	SE	t	*p*
Infrastructure and daily life		0.59	0.34	257.42***				
	Gender				0.09	0.02	3.85***	0.000
	Grade				−0.04	0.01	−3.24**	0.001
	Institution				−0.29	0.03	−9.53***	0.000
	Regional				0.12	0.02	5.07***	0.000
	Perceived goal attainment				0.40	0.02	18.48***	0.000
	Perceived competence acquisition				0.32	0.02	12.91***	0.000
Student satisfaction		0.69	0.48	386.69***				
	Gender				0.00	0.02	0.13	0.900
	Grade				−0.04	0.01	−3.23**	0.001
	Institution				0.10	0.03	3.72***	0.000
	Regional				0.17	0.02	7.87***	0.000
	Perceived goal attainment				0.56	0.02	27.17***	0.000
	Perceived competence acquisition				0.12	0.02	5.01***	0.000
	Infrastructure and daily life				0.16	0.02	9.39***	0.000

The regression model for perceived competence acquisition is identical to that reported in Table 2 and is therefore omitted here for brevity.

**Figure 3:**
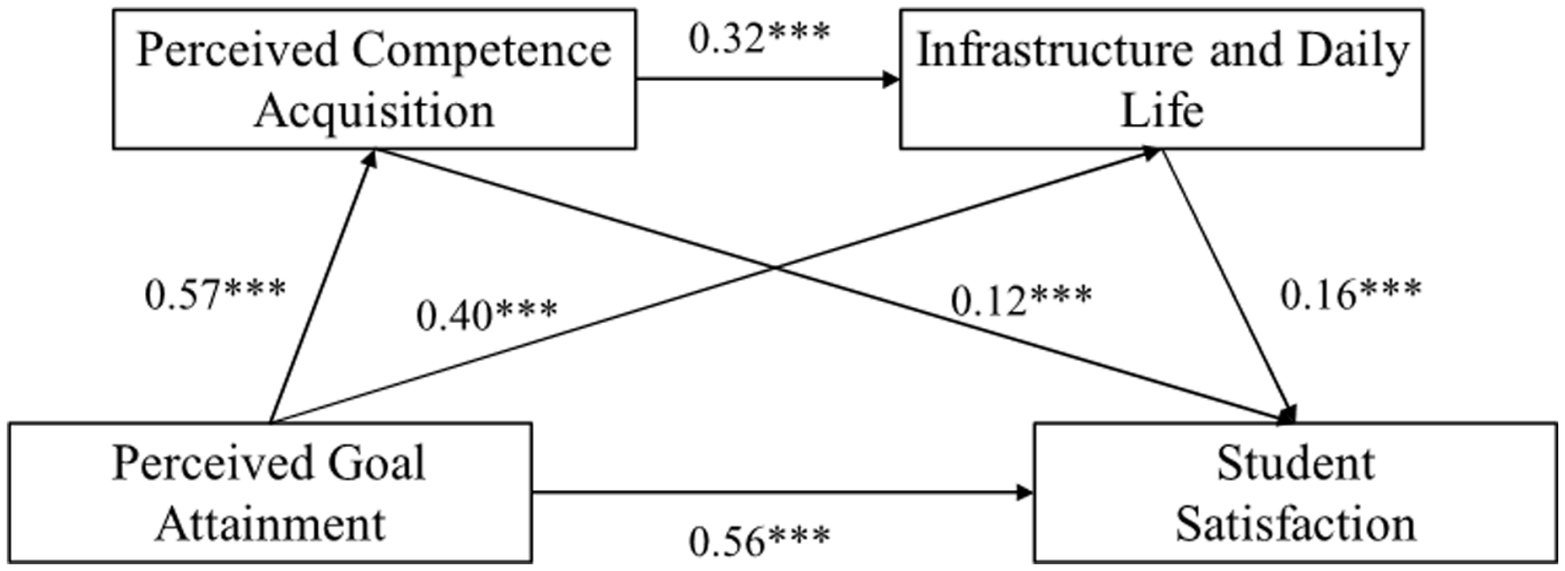
Chain mediation model linking perceived goal attainment, perceived competence acquisition, infrastructure and daily life, and student satisfaction.

As shown in Table 5 and Figure 3, both Perceived Competence Acquisition and Infrastructure and Daily Life were positively associated with Student Satisfaction (*β* = 0.12, *p* < 0.001; *β* = 0.16, *p* < 0.001), providing support for H2 and H4. Moreover, Perceived Competence Acquisition was positively associated with Infrastructure and Daily Life (*β* = 0.32, *p* < 0.001), consistent with the sequential mediation hypothesized in H6.

**Table 5:** Direct and indirect effects of perceived goal attainment on student satisfaction via perceived competence acquisition and infrastructure and daily life.

Path	Effect	SE	Bias-corrected 95%CI		Ratio
			Lower	Upper	
Total effect	0.719	0.015	0.689	0.749	100%
Direct effect	0.562	0.021	0.521	0.602	78.16%
Total indirect effect	0.157	0.021	0.117	0.196	21.84%
Indirect path1	0.066	0.019	0.029	0.104	9.18%
Indirect path2	0.062	0.01	0.043	0.083	8.62%
Indirect path3	0.029	0.005	0.019	0.040	4.03%

Note: Indirect path 1: Perceived Goal Attainment → Perceived Competence Acquisition → Student Satisfaction; Indirect path 2: Perceived Goal Attainment → Infrastructure and Daily Life → Student Satisfaction; Indirect path 3: Perceived Goal Attainment → Perceived Competence Acquisition → Infrastructure and Daily Life → Student Satisfaction.

The total effect of Perceived Goal Attainment on Student Satisfaction was 0.719, with the direct effect accounting for 78.16% (effect = 0.562, 95% CI = [0.521, 0.602]) and the total indirect effect accounting for 21.84% (effect = 0.157, 95% CI = [0.117, 0.196]). Specifically, the indirect effect of the first path “Perceived Goal Attainment → Perceived Competence Acquisition → Student Satisfaction” (Indirect Path 1) was 0.066 (95% CI = [0.029, 0.104]), accounting for 9.18% of the total effect. The indirect effect of the second path “Perceived Goal Attainment → Infrastructure and Daily Life → Student Satisfaction” (Indirect Path 2) was 0.062 (95% CI = [0.043, 0.083]), accounting for 8.62% of the total effect. The indirect effect of the third path “Perceived Goal Attainment → Perceived Competence Acquisition → Infrastructure and Daily Life → Student Satisfaction” (Indirect Path 3) was 0.029 (95% CI = [0.019, 0.040]), accounting for 4.03% of the total effect. The total indirect effect was 0.157 (95% CI = [0.117, 0.196]), indicating that the overall mediation effect path was significant.

## 5 Discussion

This study constructed a serial mediation model to examine the mechanisms through which perceived goal attainment influences student satisfaction in the Chinese higher education context. Grounded in EDT and SDT, our results confirmed the significant direct effect of goal attainment and uncovered the mediating roles of perceived competence acquisition and campus experiences (curriculum and learning, infrastructure and daily life). These findings deepen the understanding of how students’ cognitive and emotional responses shape their satisfaction and provide theoretical insights for improving the quality of learning experiences in HEIs. Compared with prior research conducted predominantly in Western contexts (e.g., Tessema et al., 2012; Pham et al., 2024), this study offers evidence from a large and diverse Chinese sample, highlighting both cross-cultural similarities and context-specific patterns in the determinants of satisfaction.

### 5.1 The direct effect of perceived goal attainment on student satisfaction

Consistent with H1, perceived goal attainment had a strong and significant direct effect on student satisfaction. This result aligns with the core tenets of EDT (Oliver, 1980), which suggests that satisfaction arises when individuals perceive a match between their expectations and actual outcomes. When students believe they are making progress toward academic or personal goals, they are more likely to evaluate their overall higher education experience positively. Prior studies have similarly found goal fulfillment to be a key predictor of satisfaction across educational settings (Tessema et al., 2012; Pham et al., 2024). Recent studies have also emphasized that perceived goal attainment serves as a cognitive resource that boosts students’ internal motivation and academic confidence (Chen et al., 2023; Zhang et al., 2024). In the context of China’s performance-driven higher education system, such perceptions may carry even greater weight, serving as a central evaluative standard in students’ assessment of their academic journey.

### 5.2 The mediating role of perceived competence acquisition

Supporting H2, perceived competence acquisition significantly mediated the relationship between goal attainment and student satisfaction. This finding is in line with SDT, which emphasizes competence as a fundamental psychological need that enhances intrinsic motivation and personal well-being (Ryan and Deci, 2000). Students who perceive that they are gaining knowledge, skills, and abilities not only experience a greater sense of self-efficacy but also derive greater meaning from their academic efforts. In this study, competence acquisition accounted for a meaningful portion of the total effect, reinforcing its explanatory importance as a psychological mechanism. This pathway highlights the internal processes through which cognitive appraisals of achievement are transformed into affective responses like satisfaction. This is consistent with findings from Vo and Ho (2024), who noted that competence acquisition promotes student satisfaction by fostering autonomy and mastery motivation.

### 5.3 The mediating role of curriculum and learning

The findings also supported H3, indicating that students’ evaluations of their academic experiences—specifically curriculum design and learning processes—partially mediated the link between perceived goal attainment and satisfaction. Although this effect was not as strong as that of competence acquisition, it remained meaningful. As students perceive greater goal progress, they may simultaneously raise their expectations for learning environments. When these expectations are met, satisfaction is further reinforced. This pattern reflects a broader psychological mechanism in which academic achievement heightens sensitivity to educational quality. Zhang et al. (2023) similarly emphasized that satisfaction with curriculum plays a key role in sustaining academic motivation.

### 5.4 The mediating role of infrastructure and daily life

The results further supported H4, showing that perceived infrastructure and daily life significantly mediated the relationship between perceived goal attainment and student satisfaction. This suggests that as students perceive higher goal attainment, they may also evaluate the adequacy of campus facilities and daily services more positively, which in turn enhances overall satisfaction. Such findings align with research indicating that quality campus infrastructure and well-organized daily services contribute substantially to the student experience and overall well-being (Guo et al., 2024; Huang and Lai, 2024).

### 5.5 The sequential mediation pathways

In addition to independent mediators, we identified two significant sequential mediation pathways. First, consistent with H5, perceived goal attainment enhanced perceived competence acquisition, which in turn improved perceptions of curriculum and learning, ultimately increasing satisfaction. Second, supporting H6, perceived goal attainment enhanced perceived competence acquisition, which then improved perceptions of infrastructure and daily life, also contributing to greater satisfaction. These findings resonate with the conservation of resources theory (Hobfoll, 2001), suggesting that students’ internal resources (such as perceived competence) can be converted into contextual engagement (such as appreciation of the learning or living environment), which cumulatively enhance satisfaction. Although these sequential effects were not the primary drivers of satisfaction, they reveal an important process of psychological reinforcement and environmental alignment.

### 5.6 Effects of control variables on student satisfaction

Regarding the control variables, several contextual factors exhibited statistically significant influences on student satisfaction, meriting further discussion. First, grade (student year) showed a consistently negative effect on satisfaction (*β* ≈ −0.03 to −0.05, *p* < 0.01), suggesting that more senior students tend to report lower satisfaction. This aligns with findings that school belonging often declines as students progress through higher grades (Neel and Fuligni, 2013; Högberg et al., 2021). Second, institution type exhibited a significant negative association with perceived infrastructure and daily life (*β* ≈ −0.29, *p* < 0.001), suggesting that students in vocational colleges reported significantly lower satisfaction with infrastructure and daily life compared to undergraduates. This is consistent with evidence that institutions with fewer resources and weaker reputations often deliver less satisfactory campus support services (Guo et al., 2024; Huang and Lai, 2024). Third, being from a more developed (regional) area was associated with higher satisfaction (*β* ≈ 0.17 to 0.19, *p* < 0.001), meaning that students from more developed regions perceived higher satisfaction. Prior Chinese higher education studies have found that institutions in developed regions tend to provide better logistical support, campus environment, and resource availability, which in turn enhance student satisfaction (Gu and Lu, 2023; Liu and Ma, 2018).

## 6 Implications and limitations

This study developed and tested a serial mediation model to explore how perceived goal attainment influences student satisfaction through competence acquisition, curriculum and learning, and infrastructure and daily life, drawing upon EDT and SDT. The findings offer both theoretical and practical implications for enhancing student experience in higher education.

From a theoretical perspective, this study expands the boundary of EDT by conceptualizing perceived goal attainment as a personal cognitive benchmark, thus extending the traditional understanding of satisfaction from expectation-outcome gaps to individual developmental evaluations. While traditional EDT emphasizes satisfaction as a function of the discrepancy between expectations and outcomes, our findings reveal that perceived goal attainment can exert a significant direct effect on satisfaction even in the absence of explicit expectation disconfirmation. This shift from a comparative to an evaluative mechanism suggests that students may anchor their satisfaction more strongly on internal perceptions of progress toward personal or institutional goals. Furthermore, this alternative pathway is particularly salient in performance-oriented and collectivist educational cultures—such as in China—where personal goal realization is often shaped by external standards and social expectations rather than individualized expectations. This adds a culturally grounded refinement to the explanatory scope of EDT. Furthermore, by integrating SDT, the model clarifies the layered psychological mechanism through which internal goal achievement is transformed into student satisfaction via competence enhancement and environmental perceptions. This dual-theory integration provides new insight into how cognitive and motivational processes interact to shape satisfaction in academic settings.

In terms of practical significance, the findings highlight several actionable pathways for improving student satisfaction. Higher education institutions should not only support students in achieving academic goals but also foster a sense of competence and provide responsive campus environments. Interventions such as formative feedback, competency-based learning models, and the optimization of learning infrastructure and support services are likely to enhance both perceived competence and overall satisfaction. These results also underscore the importance of student-centered pedagogical and service strategies.

Despite its contributions, this study has several limitations. First, it employed a cross-sectional design, which limits the ability to draw causal inferences. Future research should adopt longitudinal or experimental designs to capture the dynamic development of student perceptions and satisfaction over time. Second, the data were collected using self-report questionnaires, which may introduce biases such as social desirability or recall errors. Triangulation with behavioral data or third-party assessments could enhance measurement validity. Third, although the sample included students from 13 HEIs across different regions, convenience sampling may limit the generalizability of the findings. Broader sampling strategies and stratified designs are recommended in future studies.

Lastly, this study focused primarily on positive psychological mechanisms but did not fully explore potential moderating or contextual variables (e.g., institutional culture, academic discipline). Exploring such heterogeneity could further illuminate for whom and under what conditions goal attainment most effectively promotes satisfaction. Addressing these limitations would strengthen the robustness of future research and contribute to a more nuanced understanding of student development and well-being in diverse higher education settings.

## 7 Conclusion

This study investigated the relationship between perceived goal attainment and student satisfaction in Chinese HEIs and constructed two serial mediation models to examine the underlying mechanisms. The findings revealed that perceived competence acquisition plays a central mediating role, while curriculum and learning, as well as infrastructure and daily life, serve as important contextual pathways. Specifically, students who feel more competent are likely to report greater satisfaction with both teaching and learning experiences and daily campus environments, which in turn enhances their overall satisfaction. For Chinese higher education, this underscores the importance of fostering students’ competence development alongside continued investment in both academic and living environments. Policymakers and HEI leaders should integrate academic support and campus service improvements into quality enhancement strategies. However, the current findings should be interpreted with caution and further validated through longitudinal or cross-institutional research. Future studies could also explore how these mechanisms vary across different institutional types and cultural contexts to provide more targeted policy insights.

## Data Availability

The raw data supporting the conclusions of this article will be made available by the authors, without undue reservation.

## References

[ref1] Adler A. (1927). Understanding human nature. New York: Greenberg.

[ref2] Aldemir C. Gulcan Y. (2004). Student satisfaction in higher education: a Turkish case. High. Educ. Manag. Policy 16, 109–122. doi: 10.1787/hemp-v16-art19-en

[ref3] Alves H. Raposo M. (2007). Conceptual model of student satisfaction in higher education. Total Qual. Manag. Bus. Excel. 18, 571–588. doi: 10.1080/14783360601074315

[ref4] Alzahrani L. Seth K. P. (2021). Factors influencing students’ satisfaction with continuous use of learning management systems during the COVID-19 pandemic: an empirical study. Educ. Inf. Technol. 26, 6787–6805. doi: 10.1007/s10639-021-10492-5, PMID: 33841029 PMC8023780

[ref5] Anderson R. E. (1973). Consumer dissatisfaction: the effect of disconfirmed expectancy on perceived product performance. J. Mark. Res. 10, 38–44. doi: 10.1177/002224377301000106

[ref6] Appleton-Knapp S. L. Krentler K. A. (2006). Measuring student expectations and their effects on satisfaction: the importance of managing student expectations. J. Mark. Educ. 28, 254–264. doi: 10.1177/0273475306293359

[ref7] Arambewela R. Hall J. (2009). An empirical model of international student satisfaction. Asia Pac. J. Mark. Logist. 21, 555–569. doi: 10.1108/13555850910997599

[ref8] Bandura A. (1997). Self-efficacy: The exercise of control. New York: Cambridge University Press.

[ref9] Bao W. (2014). Measurement of college student satisfaction and its influencing factors (in Chinese). Educ. Dev. Res. 34, 22–29. doi: 10.14121/j.cnki.1008-3855.2014.03.013

[ref10] Bastian B. Kuppens P. De Roover K. Diener E. (2014). Is valuing positive emotion associated with life satisfaction? Emotion 14, 639–645. doi: 10.1037/a0036466, PMID: 24749643

[ref11] Bell K. (2021). Increasing undergraduate student satisfaction in higher education: the importance of relational pedagogy. J. Furth. High. Educ. 46, 490–503. doi: 10.1080/0309877X.2021.1985980

[ref12] Brown R. M. Mazzarol T. W. (2009). The importance of institutional image to student satisfaction and loyalty within higher education. High. Educ. 58, 81–95. doi: 10.1007/s10734-008-9183-8

[ref13] Brown S. A. Venkatesh V. Kuruzovich J. Massey A. P. (2008). Expectation confirmation: an examination of three competing models. Organ. Behav. Hum. Decis. Process. 105, 52–66. doi: 10.1016/j.obhdp.2006.09.008

[ref14] Bustos-Usta J. A. León-Gómez A. Santos-Jaén J. M. (2024). Student satisfaction: examining capacity development and environmental factors in higher education institutions. Heliyon 10:e38699. doi: 10.1016/j.heliyon.2024.e36699 PMC11402129 39281635

[ref15] Byrne B. M. (2010). Structural equation modeling with AMOS: Basic concepts, applications, and programming. 2nd Edn. New York: Routledge.

[ref16] Cardona M. M. Bravo J. J. (2012). Service quality perceptions in higher education institutions: the case of a Colombian university. Estud. Gerenc. 28, 23–29. doi: 10.1016/S0123-5923(12)70004-9

[ref1003] Cardozo R. N. (1965). An Experimental Study of Customer Effort, Expectation, and Satisfaction. Journal of Marketing Research 3, 244–249. doi: 10.2307/3150182

[ref17] Cazan A. M. Schiopca B. A. (2014). Self-directed learning, personality traits, and academic achievement. Procedia. Soc. Behav. Sci. 127, 640–644. doi: 10.1016/j.sbspro.2014.03.327

[ref18] Chadwick K. Ward J. (1987). Determinants of consumer satisfaction with education: implications for college and university administrators. Coll. Univ. 62, 236–246.

[ref19] Chen C. Bian F. Zhu Y. (2023). The relationship between social support and academic engagement among university students: the chain mediating effects of life satisfaction and academic motivation. BMC Public Health 23:2368. doi: 10.1186/s12889-023-17301-3, PMID: 38031093 PMC10688496

[ref20] Douglas J. Douglas A. Barnes B. (2006). Measuring student satisfaction at a UK university. Qual. Assur. Educ. 14, 251–267. doi: 10.1108/09684880610678568

[ref21] Douglas J. A. Douglas A. McClelland R. J. Davies J. (2015). Understanding student satisfaction and dissatisfaction: an interpretive study in the UK higher education context. Stud. High. Educ. 40, 329–349. doi: 10.1080/03075079.2013.842217

[ref22] Dugenio-Nadela C. Cañeda D. M. Tirol S. L. Samillano J. H. Pantuan D. J. M. Piañar J. C.. (2023). Service quality and students' satisfaction in higher education institutions. J. Hum. Resour. Sustain. Stud. 11, 858–870. doi: 10.4236/jhrss.2023.114049

[ref23] Elliot A. J. Dweck C. S. (2005). Handbook of competence and motivation. Guilford Publications. New York: The Guilford Press.

[ref24] Elliott K. M. Healy M. A. (2001). Key factors influencing student satisfaction related to recruitment and retention. J. Mark. High. Educ. 10, 1–11. doi: 10.1300/J050v10n0401

[ref25] Elliott K. M. Shin D. (2002). Student satisfaction: an alternative approach to assessing this important concept. J. High. Educ. Policy Manag. 24, 197–209. doi: 10.1080/1360080022000013518

[ref26] Evangelidis I. Van Osselaer S. M. J. (2018). Points of (dis)parity: expectation disconfirmation from common attributes in consumer choice. J. Mark. Res. 55, 1–13. doi: 10.1509/jmr.15.0233

[ref27] Fornell C. Larcker D. F. (1981). Structural equation models with unobservable variables and measurement error: algebra and statistics. J. Mark. Res. 18, 39–50. doi: 10.2307/3150980

[ref28] Fryer J. W. Elliot A. J. (2008). “Self-regulation of achievement goal pursuit” in Motivation and self-regulated learning: Theory, research, and applications. eds. Schunk D. H. Zimmerman B. J.. Eds ed (New York: Lawrence Erlbaum Associates Publishers), 53–75.

[ref29] Fuller C. M. Simmering M. J. Atinc G. Atinc Y. Babin B. J. (2016). Common methods variance detection in business research. J. Bus. Res. 69, 3192–3198. doi: 10.1016/j.jbusres.2015.12.008

[ref30] Gabrah A. Y. B. de Heer F. Antwi-Adjei A. (2023). Service quality affecting student satisfaction in higher education institutions in Ghana. Cogent Educ. 10:2238468. doi: 10.1080/2331186X.2023.2238468

[ref31] Gruber T. Fuß S. Voss R. Gläser-Zikuda M. (2010). Examining student satisfaction with higher education services: using a new measurement tool. Int. J. Public Sect. Manag. 23, 105–123. doi: 10.1108/09513551011022474

[ref32] Gu Q. Lu G. (2023). Factors influencing the satisfaction level of college students in China: literature analysis based on grounded theory. Front. Psychol. 13:1023420. doi: 10.3389/fpsyg.2022.1023420, PMID: 36760906 PMC9902928

[ref33] Guo T. Li T. Qi Z. (2024). Perceived school service quality and vocational students’ learning satisfaction: mediating role of conceptions of vocational education. PLoS One 19:e0307392. doi: 10.1371/journal.pone.0307392, PMID: 39167617 PMC11338464

[ref34] Hair J. F. Babin B. J. Black W. C. Anderson R. E. (2018). Multivariate data analysis. 8th Edn. Boston, MA: Cengage Learning.

[ref1002] Hair J. F. Anderson R. E. Tatham R. L. Black W. C. (1998). Multivariate data analysis. (5th ed). Upper Saddle River, NJ: Prentice Hall.

[ref35] Hayes A. F. (2013). Introduction to mediation, moderation, and conditional process analysis: A regression-based approach. New York: Guilford Press.

[ref36] Heidenreich S. Wittkowski K. Handrich M. Falk T. (2015). The dark side of customer co-creation: exploring the consequences of failed co-created services. J. Acad. Mark. Sci. 43, 279–296. doi: 10.1007/s11747-014-0387-4

[ref37] Hobfoll S. E. (1989). Conservation of resources: a new attempt at conceptualizing stress. Am. Psychol. 44, 513–524. doi: 10.1037/0003-066X.44.3.513, PMID: 2648906

[ref38] Hobfoll S. E. (2001). The influence of culture, community, and the nested-self in the stress process: advancing conservation of resources theory. Appl. Psychol. 50, 337–421. doi: 10.1111/1464-0597.00062

[ref39] Högberg B. Petersen S. Strandh M. Johansson K. (2021). Determinants of declining school belonging 2000–2018: the case of Sweden. Soc. Indic. Res. 157, 783–802. doi: 10.1007/s11205-021-02662-2

[ref40] Hu Y. (2018). Influencing factors of learning satisfaction among students in local undergraduate colleges: from the perspective of students’ self-learning efficacy (in Chinese). High. Educ. Expl. 3, 43–50. Available at: https://kns.cnki.net/kcms2/article/abstract?v=wScU5_zS5COeGlNwuO-Li5pN8hIBo0BBc24uowjQRNVb9nH0KcNZu6wAOBHkg-mwvuuC0i4Dp4SBQDbjWVPsqhGdvYLod8NOqKo--XeSfmGEiAUc9GiawibhjUGPmoON1SWLDYPXkeZsCyaKQBV2h_KpnYS5cJgKc2VG5tN6xro=&uniplatform=NZKPT (Accessed July 7, 2025).

[ref41] Huang R. Lai P. C. (2024). A study on the factors influencing student satisfaction in vocational college based on the customer satisfaction theory model. Adv. Vocat. Tech. Educ. 6:215. doi: 10.23977/avte.2024.060215

[ref42] Ikram M. Kenayathulla H. B. (2023). Education quality and student satisfaction nexus using instructional material, support, classroom facilities, equipment, and growth: higher education perspective of Pakistan. Front. Educ. 8:1140971. doi: 10.3389/feduc.2023.1140971

[ref43] Jiménez-Bucarey C. Acevedo-Duque Á. Müller-Pérez S. Aguilar-Gallardo L. Mora-Moscoso M. Vargas E. C. (2021). Student’s satisfaction of the quality of online learning in higher education: an empirical study. Sustainability 13:11960. doi: 10.3390/su132111960

[ref44] Kandiko Howson C. B. Mawer M. (2013). Student expectations and perceptions of higher education. London, UK: King's Learning Institute.

[ref45] Kanwar A. Sanjeeva M. (2022). Student satisfaction survey: a key for quality improvement in the higher education institution. J. Innov. Entrepreneurship 11:27. doi: 10.1186/s13731-022-00196-6

[ref1004] Khan J. Hemsley-Brown J. (2021). Student satisfaction: the role of expectations in mitigating the pain of paying fees. J. Mark. High. Educ. 34, 178–200. doi: 10.1080/08841241.2021.1973646

[ref46] Khan J. Hemsley-Brown J. (2024). Student satisfaction: The role of expectations in mitigating the pain of paying fees. Journal of Marketing for Higher Education, 34, 178–200. Available at: https://eric.ed.gov/?redir=http%3a%2f%2fdx.doi.org%2f10.1080%2f08841241.2021.1973646

[ref47] Kline R. B. (2015). Principles and practice of structural equation modeling. 4th Edn. New York: The Guilford Press.

[ref48] Komarraju M. Karau S. J. Schmeck R. R. (2010). Role of the big five personality traits in predicting college students' academic motivation and achievement. Learn. Individ. Differ. 19, 47–52. doi: 10.1016/j.lindif.2008.07.001

[ref49] Kuh G. D. Kinzie J. L. Buckley J. A. Bridges B. K. Hayek J. C. (2006). What matters to student success: a review of the literature. Available at: https://nces.ed.gov/npec/pdf/kuh_team_report.pdf (Accessed July 5, 2025).

[ref50] Letcher D. W. Neves J. S. (2010). Determinants of undergraduate business student satisfaction. Res. High. Educ. J. 6, 1–26. Available online at: https://api.semanticscholar.org/CorpusID:2373494 (Accessed July 7, 2025).

[ref53] Liu W. H. Ma R. (2018). Regional inequality of higher education resources in China. Front. Educ. China 13, 119–151. doi: 10.1007/s11516-018-0005-1

[ref1009] Li Y. (2017). Research on student satisfaction in higher education based on structural equation modeling (in Chinese). High. Educ. Expl 2, 45–50. Available online at: https://kns.cnki.net/kcms2/article/abstract?v=wScU5_zS5COcqEIX7nVCyMnCTvQRyp3bEn2y3yD3NcxHZ5jPqdDxioPYh3I3ajy904Cja8mlZfIJruiY9xgSg_udzURuAg360-v772IjCT-DcjWrkYIqf4bR7ym_gdcx9K-TkTPF7FWlbxcOuX4xL7I-0cDtgIyUVR0JJEZTW8k=&uniplatform=NZKPT (Accessed July 7, 2025).

[ref55] Locke E. A. Latham G. P. (2002). Building a practically useful theory of goal setting and task motivation: a 35-year odyssey. Am. Psychol. 57, 705–717. doi: 10.1037/0003-066X.57.9.705, PMID: 12237980

[ref56] Luna-Cortes G. (2024). Managing students’ illusion of control in higher education: effect on unrealistic optimism and expectancy disconfirmation. Higher Educ. 88, 2187–2204. doi: 10.1007/s10734-024-01212-2

[ref57] Maniriho A. (2024). Satisfaction and academic performance of undergraduate economics students. Cogent Educ. 11:707. doi: 10.1080/2331186X.2024.2326707

[ref58] Maslow A. H. (1954). Motivation and personality. New York: Harper & Row.

[ref59] Menard S. (1995). Applied logistic regression analysis. Sage University paper series on quantitative applications in the social sciences, series no 07-106. Thousand Oaks, CA: Sage.

[ref60] Neel C. G. Fuligni A. (2013). A longitudinal study of school belonging and academic motivation across high school. Child Dev. 84, 678–692. doi: 10.1111/j.1467-8624.2012.01862.x, PMID: 23002809

[ref61] Nguyen H. V. Vu T. D. Saleem M. Yaseen A. (2024). The influence of service quality on student satisfaction and student loyalty in Vietnam: the moderating role of the university image. J. Trade Sci. 12, 37–59. doi: 10.1108/JTS-12-2023-0032

[ref62] Niedrich R. W. Kiryanova E. Black W. C. (2005). The dimensional stability of the standards used in the disconfirmation paradigm. J. Retail. 81, 49–57. doi: 10.1016/j.jretai.2005.01.005

[ref63] Oliver R. L. (1980). A cognitive model of the antecedents and consequences of satisfaction decisions. J. Mark. Res. 17, 460–469. doi: 10.1177/002224378001700405

[ref64] Oliver R. L. (2010). Satisfaction: A behavioral perspective on the consumer. 2nd Edn. Armonk, New York: M.E. Sharpe.

[ref65] Oliver R. L. Burke R. R. (1999). Expectation processes in satisfaction formation: a field study. J. Serv. Res. 1, 196–214. doi: 10.1177/109467059913002

[ref66] Olshavsky R. W. Miller J. A. (1972). Consumer expectations, product performance and perceived product quality. J. Mark. Res. 9, 19–21. doi: 10.2307/3149600

[ref67] Pham T. T. H. Ho T. T. Q. Nguyen B. T. N. Nguyen H. T. Nguyen T. H. (2024). Academic motivation and academic satisfaction: a moderated mediation model of academic engagement and academic self-efficacy. J. Appl. Res. High. Educ. 16, 1999–2012. doi: 10.1108/JARHE-10-2023-0474

[ref68] Podsakoff P. M. MacKenzie S. B. Lee J. Y. Podsakoff N. P. (2003). Common method biases in behavioral research: a critical review of the literature and recommended remedies. J. Appl. Psychol. 88, 879–903. doi: 10.1037/0021-9010.88.5.879, PMID: 14516251

[ref69] Rutkowski L. Svetina D. (2014). Assessing the hypothesis of measurement invariance in the context of large-scale international surveys. Educ. Psychol. Meas. 74, 31–57. doi: 10.1177/0013164413498257

[ref70] Ryan R. M. Deci E. L. (2000). Self-determination theory and the facilitation of intrinsic motivation, social development, and well-being. Am. Psychol. 55, 68–78. doi: 10.1037/0003-066X.55.1.68, PMID: 11392867

[ref71] Ryan R. M. Deci E. L. (2020). Intrinsic and extrinsic motivation from a self-determination theory perspective: definitions, theory, practices, and future directions. Contemp. Educ. Psychol. 61:101860. doi: 10.1016/j.cedpsych.2020.101860

[ref72] Schunk D. H. Pajares F. (2009). “Self-efficacy theory” in Handbook of motivation at school. eds. Wentzel K. R. Wigfield A.. Eds ed (New York: Routledge), 35–53.

[ref73] Soomro K. A. Kale U. Curtis R. Akcaoglu M. Bernstein M. (2020). Digital divide among higher education faculty. Int. J. Educ. Technol. High. Educ. 17, 1–16. doi: 10.1186/s41239-020-00191-5 PMC12459395 41001069

[ref74] Spreng R. A. MacKenzie S. B. Olshavsky R. W. (1996). A reexamination of the determinants of consumer satisfaction. J. Mark. 60, 15–32. doi: 10.2307/1251839

[ref1005] Spreng R. A. Page T. J. (2003). A test of alternative measures of disconfirmation. Decision Sciences, 34, 31–62. doi: 10.1111/1540-5915.02214

[ref75] Suarman N. V. Aziz Z. Yasin R. M. (2013). The quality of teaching and learning towards the satisfaction among the university students. Asian Soc. Sci. 9, 252–260. doi: 10.5539/ass.v9n12p252

[ref76] Surya Bahadur G. C. Gurung S. K. Poudel R. L. Yadav U. S. Bhattacharjee A. Dhungana B. R. (2024). The effect of higher education service quality on satisfaction among business students in India and Nepal. Cogent Educ. 11:521. doi: 10.1080/2331186X.2024.2393521

[ref77] Taylor A. B. MacKinnon D. P. Tein J. Y. (2008). Tests of the three-path mediated effect. Organ. Res. Methods 11, 241–269. doi: 10.1177/1094428107300344

[ref78] Tessema M. T. Ready K. Yu W. (2012). Factors affecting college students’ satisfaction with major curriculum: evidence from nine years of data. Int. J. Humanit. Soc. Sci. 2, 34–44. Available at: https://www.ijhssnet.com/journals/Vol_2_No_2_Special_Issue_January_2012/5.pdf (Accessed July 7, 2025).

[ref79] Trowler V. (2010). Student engagement: A literature review. New York, UK: The Higher Education Academy.

[ref80] Umar M. Hasan M. (2024). Predicting students’ satisfaction with academic services at a multicultural engineering university in Bangladesh: a multiple regression analysis. PLoS One 19:e0309223. doi: 10.1371/journal.pone.0309223, PMID: 39240927 PMC11379188

[ref81] Van Dinther M. Dochy F. Segers M. (2011). Factors affecting students’ self-efficacy in higher education. Educ. Res. Rev. 6, 95–108. doi: 10.1016/j.edurev.2010.10.003

[ref82] Van Ryzin G. G. (2004). Expectations, performance, and citizen satisfaction with urban services. J. Policy Anal. Manage. 23, 433–448. doi: 10.1002/pam.20020

[ref83] Van Ryzin G. G. (2006). Testing the expectancy disconfirmation model of citizen satisfaction with local government. J. Public Adm. Res. Theory 16, 599–611. doi: 10.1093/jopart/mui058

[ref84] Van Ryzin G. G. (2013). An experimental test of the expectancy-disconfirmation theory of citizen satisfaction. J. Policy Anal. Manage. 32, 597–614. doi: 10.1002/pam.21702

[ref85] Vo H. Ho H. (2024). Online learning environment and student engagement: the mediating role of expectancy and task value beliefs. Aust. Educ. Res. 51, 2183–2207. doi: 10.1007/s13384-024-00689-1

[ref86] Wang H. Guo J. (2020). An analysis of students’ sense of gain in employment and entrepreneurship education in higher vocational colleges: a survey focusing on students’ satisfaction with employment and entrepreneurship courses (in Chinese). Vocat. Tech. Educ. 41, 60–65. Available at: https://kns.cnki.net/kcms2/article/abstract?v=wScU5_zS5COZOYzbH2VcycIGKwzWl3NSLme_qMI_coB-vKV_ARcBW3gGOhvAC1oR8YoWxBTiB_DZ1Zrm4i6C6C6p-Qq6265xcIEmq3khAxe45uQxrH4x4gFZDv5kPKU97Ink5_07PYelMUNCZBKGa_YnLGGXYgdYiE1rZbx1eBE=&uniplatform=NZKPT (Accessed July 5, 2025).

[ref88] Wang J. Liu X. (2019). Current situation, changes, and interrelations: sense of security, sense of gain, and sense of happiness and their improvement paths (in Chinese). Jiangsu Soc. Sci. 258, 41–49. doi: 10.13858/j.cnki.cn32-1312/c.2019.01.006

[ref90] Wong W. H. Chapman E. (2023). Student satisfaction and interaction in higher education. High. Educ. 85, 957–978. doi: 10.1007/s10734-022-00874-0, PMID: 35669591 PMC9159046

[ref89] Wiers-Jenssen J. Stensaker B. Grøgaard J. B. (2002). Student satisfaction: towards an empirical deconstruction of the concept. Qual. High. Educ. 8, 183–195. doi: 10.1080/1353832022000004377

[ref91] Xu C. (2017). Evaluation of service quality in graduate education and analysis of its influencing factors (in Chinese). High. Educ. Dev. Eval. 33, 30–49. 114–115. doi: 10.3963/j.issn.1672-8742.2017.05.004

[ref92] Yang D. Chen P. Wang H. Wang K. Huang R. (2022). Teachers' autonomy support and student engagement: a systematic literature review of longitudinal studies. Front. Psychol. 13:925955. doi: 10.3389/fpsyg.2022.925955, PMID: 36072024 PMC9441875

[ref93] Zeithaml V. A. (1988). Consumer perceptions of price, quality, and value: a means–end model and synthesis of evidence. J. Mark. 52, 2–22. doi: 10.1177/002224298805200302

[ref94] Zhang J. Hu Z. Zheng S. Li Y. Wang Y. Chen L. (2023). A chain mediation model reveals the association between mindfulness and depression of college students. Sci. Rep. 13:16830. doi: 10.1038/s41598-023-43984-0 37803146 PMC10558579

[ref95] Zhang X. Li Y. Wang M. (2024). Chain mediation effects in educational satisfaction research: a meta-analytic review. Educ. Psychol. Rev. 36, 123–145. doi: 10.1007/s10648-023-09876-5

[ref96] Zhang C. Liao H. Yang Q. Liang L. (2022). The expectancy-disconfirmation model and citizen satisfaction with public services: a meta-analysis and an agenda for best practice. Public Adm. Rev. 82, 147–159. doi: 10.1111/puar.13368

[ref97] Zhang B. Lin J. (2014). An empirical analysis of factors affecting college teaching satisfaction: from the perspective of student expectations and perceived quality (in Chinese). Fudan Educ. Forum 12, 59–65. doi: 10.13397/j.cnki.fef.2014.04.011

[ref98] Zhang Q. Yue C. (2009). Higher education quality evaluation and student satisfaction (in Chinese). China High. Educ. Research 11, 40–43. doi: 10.16298/j.cnki.1004-3667.2009.11.012

[ref1001] Zhang H. Foskett N. Wang D. Qu M. (2011). Student satisfaction with undergraduate teaching in China—A comparison between research-intensive and other universities. Higher Education Policy, 24, 1–24. doi: 10.1057/hep.2010.23

